# The Effects of Graphene Oxide Nanoparticles on the Cryopreservation of Angora Buck Sperm

**DOI:** 10.3390/molecules31060955

**Published:** 2026-03-12

**Authors:** Ali Erdem Öztürk, Mustafa Bodu, Yunus Emre Atay, Serpil Sarıözkan, Derya Şahin, Oya Korkmaz, İsmail Öçsoy, Mustafa Hitit

**Affiliations:** 1 Department of Reproduction and Artificial Insemination, Faculty of Veterinary Medicine, Erciyes University, Kayseri 38280, Türkiye; serpilsariozkan@erciyes.edu.tr; 2 Department of Reproduction and Artificial Insemination, Faculty of Veterinary Medicine, Selcuk University, Konya 42130, Türkiye; mbodu@selcuk.edu.tr; 3 Department of Obstetrics and Gynecology, Faculty of Veterinary Medicine, Erciyes University, Kayseri 38280, Türkiye; yunusemreatay@erciyes.edu.tr; 4 International Livestock Research and Training Center, Ankara 06270, Türkiye; vh.sahinderya@icloud.com; 5 Department of Histology and Embryology, Faculty of Medicine, Malatya Turgut Özal University, Malatya 44210, Türkiye; oya.korkmaz@ozal.edu.tr; 6 Department of Analytical Chemistry, Faculty of Pharmacy, Erciyes University, Kayseri 38280, Türkiye; ismailocsoy@erciyes.edu.tr; 7 Cooperative Agricultural Research Center, College of Agriculture, Food, and Natural Resources, Prairie View A&M University, Prairie View, TX 77446, USA

**Keywords:** nano-graphene oxide, angora buck, sperm, cryopreservation, nanoparticle

## Abstract

Nano-graphene oxide (NGO) is a nanomaterial that has been frequently used in the fields of health and bioengineering in recent years. However, its potential use in semen cryopreservation is still in the exploratory phase. In this study, Angora bucks, a breed with low resistance to cold shock, were used. Sperm was collected from five different Angora bucks, pooled, diluted with a Tris-based egg yolk diluent, and frozen with the addition of NGO at two different sizes (50 and 500 nm) and doses (10 and 50 µg/mL). Nanoparticle characterization was performed using field emission scanning electron microscopy (FE-SEM), dynamic light scattering (DLS), and Fourier-transform infrared spectroscopy (FTIR). Post-thaw sperm analyses were evaluated based on motility and kinematic parameters, mitochondrial membrane potential (MMP), plasma membrane and acrosome integrity (PMAI), and DNA fragmentation. Applying 50 nm NGO at a dose of 50 µg/mL led to statistically significant improvements in motility and PMAI (*p* < 0.05). The same dose of 500 nm NGO, however, only showed a statistically significant improvement in the PMAI parameter (*p* < 0.05). No significant differences were observed between the groups for MMP and kinematic parameters (*p* > 0.05). Conversely, it was found that all sizes and doses of NGO significantly protected post-thaw sperm regarding DNA integrity (*p* < 0.05). These findings indicate that the NGO, at a size of 50 nm and a dose of 50 µg/mL, improves the post-thaw quality of Angora buck sperm and provides a cryoprotective effect that depends on size and dose. This study provides preliminary data on the potential effects of NGO; however, comprehensive mechanistic and in vivo validation studies are required to establish the biological and clinical validity of these findings.

## 1. Introduction

The Angora goat is one of the world’s critical genetic resources and has economic value through mohair production. The preservation of this breed’s genetic diversity and the inheritance of its genetic characteristics to future generations are possible through semen freezing. Semen cryopreservation is critical for the development of artificial insemination programs, the preservation of genetic resources, and the reduction in disease-spreading risk. Consequently, researchers have performed multiple investigations on the cryopreservation and storage of semen from Angora bucks [1,2].

Spermatozoa are highly susceptible to damage during freezing due to several factors, primarily cold shock, ice crystal formation, and oxidative stress. Lipid phase transitions in the plasma membrane during rapid cooling cause cold shock, disrupting membrane fluidity and leading to decreased sperm motility and fertilization capacity [3,4]. In addition, calcium ion leakage and an increase in reactive oxygen species (ROS) during freezing cause cellular damage via lipid peroxidation and mitochondrial dysfunction in sperm cells [5,6]. The damage occurring in the cell differs between rapid and slow freezing. During cryopreservation, intracellular ice forms in rapidly frozen sperm cells, causing mechanical damage. When the medium is cooled slowly, extracellular ice forms in the unfrozen portion, which has low osmotic pressure, entrapping spermatozoa and causing shrinkage and damage to the cell membrane. This condition is referred to as the two-factor hypothesis [7]. Traditionally, intracellular ice formation during rapid cooling and solution effects in slow cooling have been considered the primary causes of damage; however, recent studies suggest that in sperm cells, the damage caused by rapid cooling is mainly due to osmotic imbalance during thawing [8]. In addition to this information, goat semen (especially from Angora bucks) is more vulnerable to lipid peroxidation damage due to its high polyunsaturated fatty acid (PUFA) content [9]. The numerous double bonds in PUFAs react with ROS, disrupting membrane integrity and leading to acrosome damage, mitochondrial dysfunction, and loss of motility. Consequently, current studies are also being conducted to increase the freezeability of goat sperm [10].

To prevent this damage, various methods have been developed. Traditional cryoprotectants (such as glycerol and DMSO), slow freezing, rapid freezing, vitrification, and rapid warming techniques are used to reduce ice formation and related damage [11]. However, recent studies have reported that magnetic nanoparticles alter the freezing temperature of ice and act as nucleation centers for ice [12]. In another study, it was reported that magnetic nanoparticles act as ice nucleation centers and that magnetic fields facilitate supercooling [13]. In a further analysis, the effects of metallic nanomaterials (especially zinc oxide and selenium nanoparticles) on post-cryopreservation sperm quality, lipid peroxidation, sperm head ultrastructure, and field fertility were evaluated; the results showed that these nanoparticles preserve sperm morphology and may increase fertilization rates by reducing oxidative damage [14].

Graphene oxide (GO) is a nanomaterial that has recently emerged in reproductive studies. GO has been attracting increasing interest in the biomedical field and has various potential applications in reproductive biotechnologies. In particular, the positive effects of GO are being investigated in processes such as cryopreservation, sperm capacitation, and in vitro fertilization (IVF). Singh et al. (2022) found in their study on bovine and buffalo sperm that GO at concentrations of 0.05–0.1 mg/mL increased sperm motility, viability, and membrane integrity [15]. GO supports capacitation by removing cholesterol from the sperm membrane, thereby increasing fertilization capacity. However, toxic effects have also been observed at high doses [16]. A toxicology study conducted on human sperm demonstrated that low doses of GO did not impair viability or cause oxidative stress. Furthermore, no adverse effects have been observed in sperm sorting systems [17]. In a rat study, high doses of GO were reported to cause testicular tissue damage, whereas low and medium doses were safe [18]. It is also stated that GO can increase capacitation and, consequently, fertilisation by removing membrane cholesterol, and therefore can be used within the scope of assisted reproductive technologies [19].

However, there are some unexplored areas regarding the use of NGO in semen cryopreservation. These include studies such as determining the optimal NGO concentration for different species, understanding the protective mechanisms in detail, and developing standardized protocols. At the same time, most studies have focused on mouse and human sperm, and only one study published very recently investigated the effectiveness of NGO on small ruminant sperm [20]. Therefore, this study aimed to address a gap in the literature by examining the effects of freezing Angora buck semen at two doses and two sizes with NGO on post-thaw spermatological parameters.

## 2. Results

### 2.1. Characterization Results

**Figure 1:**
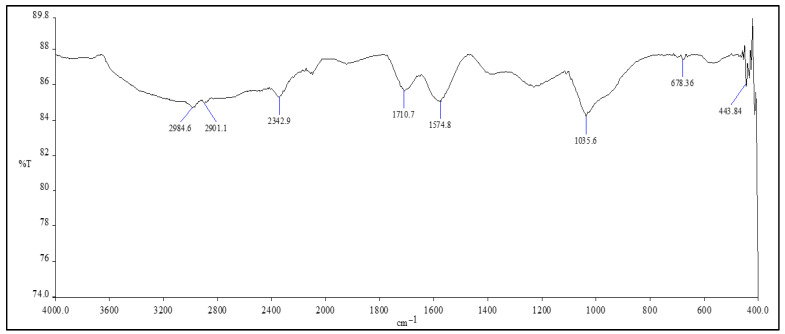
FTIR spectrum of an NGO with a size of 500 nm. Since the 50 nm NGO was obtained by sonication at 500 nm, no additional FTIR analysis was required.

For both sizes, the FTIR spectra were similar. When the FTIR spectrum of 500 nm NGO was interpreted, the stretching vibration peak of C–O bonds was observed at approximately 1035.6 cm^−1^. The vibration peak of the carbonyl group can be attributed to 1710 cm^−1^. A weak stretching peak was observed at approximately 2984.6 cm^−1^ and 2901 cm^−1^, which can be attributed to O–H and C–H groups (Figure 1).

**Figure 2:**
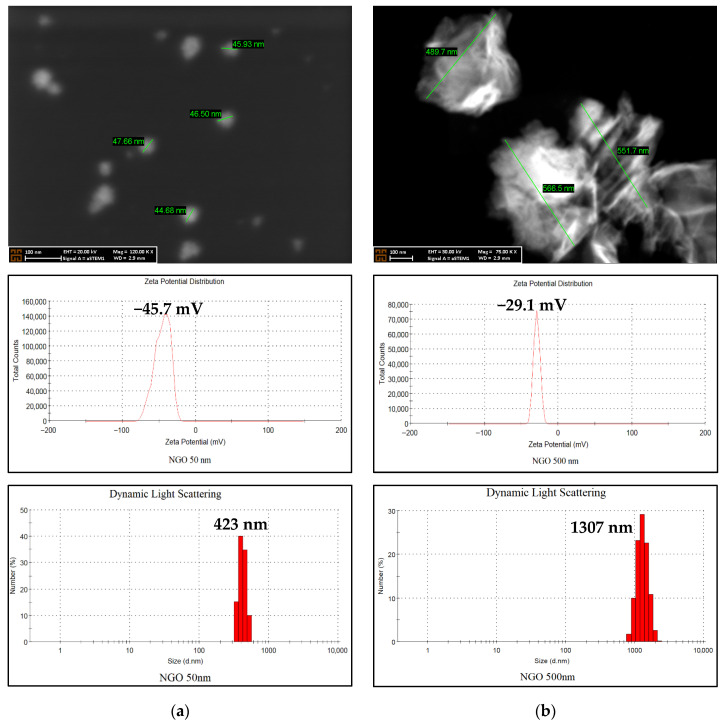
FE-SEM, Zeta potential, and DLS results of 50 nm NGO and 500 nm NGO. (**a**) Characterization data for the 50 nm NGO (top to bottom). (**b**) Characterization data for the 500 nm NGO (top to bottom).

Upon examination of the FE-SEM images, it was observed that the core sizes of the NGO nanoparticles converged around approximately 50 nm and 500 nm, achieving the targeted nanoparticle size for the study. When the zeta potential data were evaluated, 50 nm NGO and 500 nm NGO yielded values of −45.7 mV and −29.1 mV, respectively. DLS determines the hydrodynamic diameters of nanoparticles in solution by analyzing their Brownian motion. The hydrodynamic size of NGO, observed as 50 nm in FE-SEM images, was measured at approximately 423 nm, while that of NGO, observed as 500 nm, was measured at approximately 1307 nm (Figure 2).

### 2.2. Motility and Kinematic Analysis

**Figure 3:**
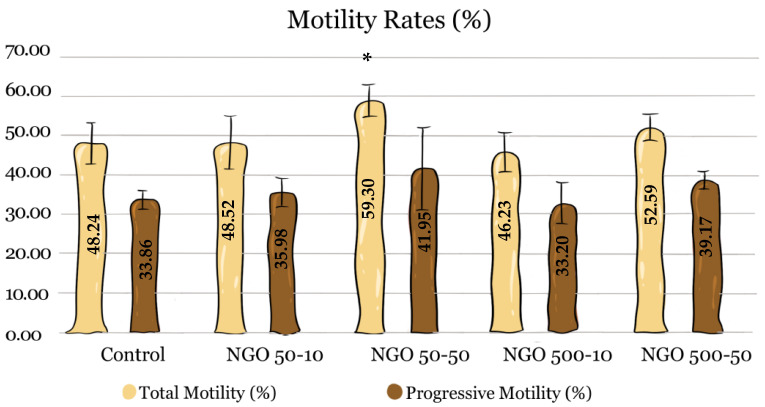
Total and progressive motility results. Asterisk indicates a statistically significant difference between the experimental groups and the control (n = 5, *p* < 0.05). Data are presented as mean ± SEM.

When total motility rates were examined, a statistically significant difference between groups was observed only in the NGO 50-50 group. In this group, the total motility rate was determined to be 59.30%, which is significantly higher than that of the control group (48.24%) (*p* < 0.05). No statistically significant difference was found in the NGO 50-10 (48.52%), NGO 500-10 (46.23%) and NGO 500-50 (52.59%) groups compared to the control group (n = 5, *p* > 0.05) (Figure 3).

When progressive motility rates were examined, numerical increases were observed in the NGO 50-50 (41.95%) and NGO 500-50 (39.17%) groups compared with the control group (33.86%). However, no statistically significant difference was determined between the groups (Figure 3).

**Figure 4:**
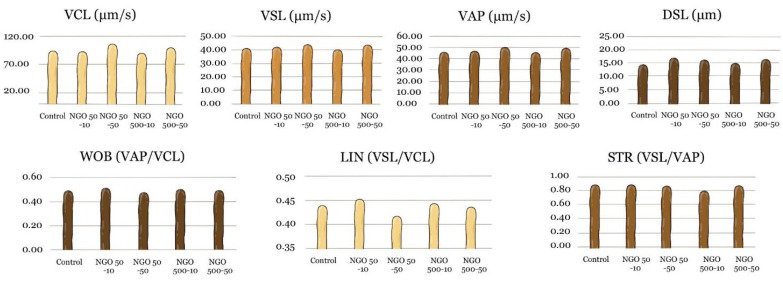
Graphs of kinematic data. When evaluating sperm motility characteristics, no statistically significant difference was observed between groups (n = 5, *p* > 0.05).

Regarding the kinematic parameters (VCL, VSL, VAP, DSL, LIN, STR, and WOB), numerically higher values were observed in the NGO 50-50 and NGO 500-50 groups compared to the control group. However, no statistically significant difference was detected between the groups for any of these parameters (Figure 4, n = 5, *p* > 0.05).

### 2.3. Flow Cytometric Analysis

**Figure 5:**
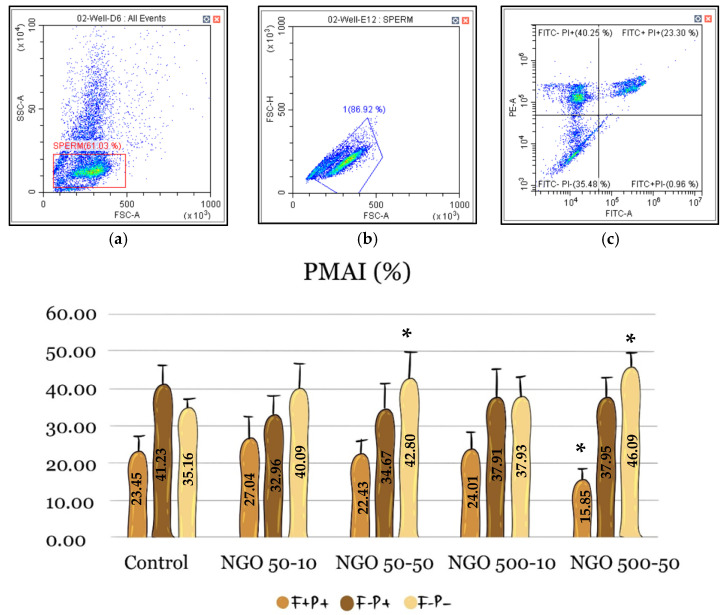
Plasma membrane and acrosomal integrity rates. The F+P+ indicates the rate of spermatozoa with both acrosomal and plasma membrane damage, F−P+ suggests the rate of spermatozoa with intact acrosome but damaged membrane, and F-P- indicates the rate of spermatozoa with both intact acrosome and plasma membrane. Asterisk indicates a statistically significant difference between the experimental groups and the control (n = 5, *p* < 0.05). Data are presented as mean ± SEM. Panels (**a**,**b**) show sperm gating, while panel (**c**) illustrates the distribution of spermatozoa based on membrane and acrosome status. FSC-A indicates cell size; SSC-A reflects internal complexity; FSC-H helps differentiate single cells from doublets.

Flow cytometry analyses consisted of two parameters: PMAI and MMP. In the PMAI data, where plasma membrane and acrosome integrity were evaluated together, the proportion of spermatozoa with both intact plasma membrane and acrosome (F−P−) was 35.16% in the control group. Although the F−P−value was higher in all groups, statistically significant protection was observed only in the NGO 50-50 (42.80%) and NGO 500-50 (46.09%) groups compared to the control group (n = 5, *p* < 0.05) (Figure 5).

**Figure 6:**
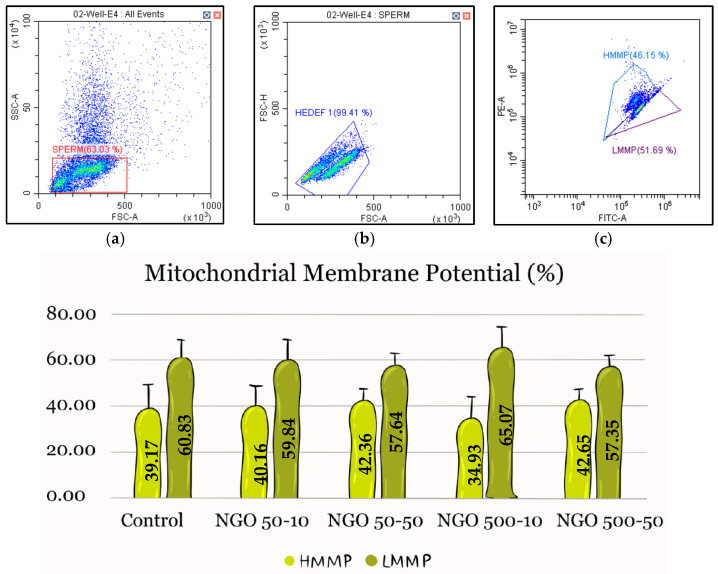
High and low MMP data. There is no statistically significant difference between the groups for HMMP and LMMP (n = 5, *p* > 0.05). Data are presented as mean ± SEM. Panels (**a**,**b**) show sperm gating, while panel (**c**) illustrates the distribution of spermatozoa based on MMP. FSC-A indicates cell size; SSC-A reflects internal complexity; FSC-H helps differentiate single cells from doublets.

When MMP data were examined, the highest HMMP was observed in the NGO 500-50 group (42.65%), while the lowest HMMP was observed in the NGO 500-10 group (34.93%). The control group showed an HMMP rate of 39.17%. The other groups were ranked within this range, and no statistically significant difference was found between the groups (n = 5, *p* > 0.05) (Figure 6).

### 2.4. DNA Fragmentation

**Figure 7:**
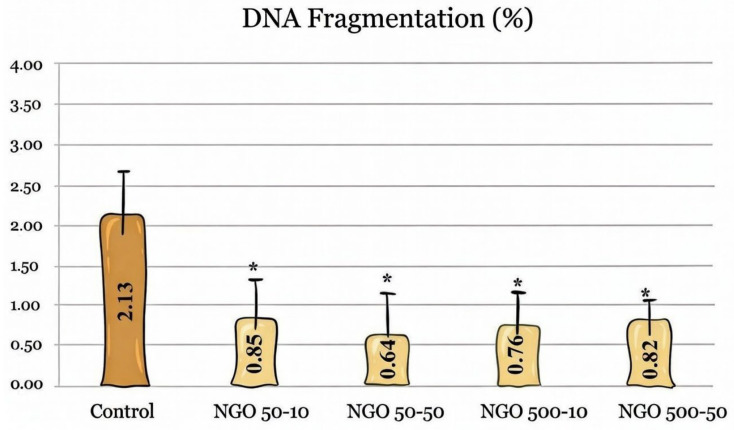
DNA fragmentation rates between groups. All groups with NGOs showed significantly lower DNA fragmentation than the control group (n = 5, *p* < 0.05). Asterisk indicates a statistically significant difference between the experimental groups and the control. Data are presented as mean ± SEM.

When DNA fragmentation results were evaluated, the DNA fragmentation rate across all NGO-containing groups ranged from 0.64% to 0.85%. The rate in the control group was 2.13%. Across all doses and sizes, the NGO showed statistically significantly lower values than the control group (n = 5, *p* < 0.05), while no significant differences were observed among the NGO groups themselves (Figure 7).

## 3. Discussion

In this study, the effects of two different NGO sizes (50 and 500 nm) and doses (10 and 50 µg/mL) on post-sperm quality in Angora buck semen were investigated. In this context, the focus was on motility, kinematic parameters, PMAI, MMP, and DNA integrity.

Characterization results showed that the zeta potential of the 50 nm NGO (−45.7 mV) was more negative than that of the 500 nm NGO (−29.1 mV). A negative zeta potential indicates that small NGOs have higher surface charge and stability [21]. This is due to the ionization of oxygen-containing functional groups on the surfaces of small nanoparticles, and similar findings have been reported in the literature [22,23,24]. The difference in zeta potential observed in our study may be due to indirect ionization of surface functional groups induced by the sonication environment during the size adjustment processes.

In FE-SEM analyses, the particle sizes of NGOs were approximately 50 nm and 500 nm, whereas in DLS analyses, they were approximately 423 nm and 1307 nm, respectively. This difference can be explained by the water retention of oxygen-containing functional groups on NGO surfaces in aqueous environments and by the formation of small particle clusters [25,26,27,28].

In this study, toxicity was not directly evaluated. However, in the literature, well-purified nanoscale graphene oxide has been reported to exhibit low cytotoxicity in studies conducted on different cell lines, including A549, KB, Raji, HCT-116, OVCAR-3, U87MG, and MCF-7, at a dose range of 1–200 µg/mL [29,30,31]. These findings show that the biological effects of graphene oxide are largely dependent on dose, particle size, surface chemistry, and impurity levels, but no significant toxic effects were observed in cell lines other than sperm at the doses used in our study.

When total motility and progressive motility data were evaluated, statistically significant protection was observed only in the NGO 50-50 group compared to the control group. In the PMAI data, sperm membrane integrity was significantly preserved in the NGO 50-50 and NGO 500-50 groups. Regarding DNA integrity, significantly lower DNA damage was observed in all NGO groups compared to the control group. No statistically significant differences were observed between the groups for MMP and kinematic parameters.

**Figure 8:**
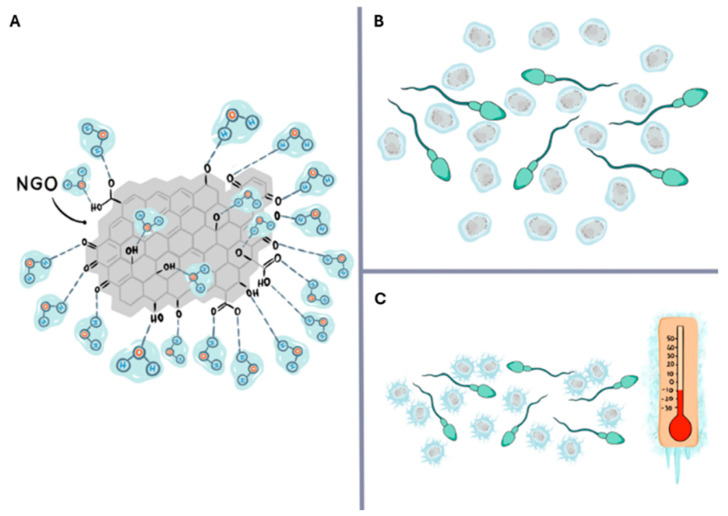
Visualization of the hypothesis that the NGO acts as a water-binding capacity and ice nucleation center. The high-oxygen functional groups on the surface of the NGO and its water retention property (**A**). The bound water acts as a nucleation center for ice formation during sperm freezing (**B**,**C**).

The statistically significant but limited protection observed in total motility data in the NGO 50-50 group is similar to results reported for bovine and buffalo [15], ram [20], and equine [32] sperm. These studies suggest that the increase in motility is due to the ice-inhibiting effect of NGOs. One of the key determinants of cell damage during sperm cryopreservation is ice crystal formation [33]. Intracellular and extracellular ice crystals exert mechanical stress, disrupting membrane integrity and causing cellular structural damage [34]. This structural damage also indirectly affects motility. It has been reported in the literature that NGOs containing oxygen-rich functional groups such as carboxyl, hydroxyl, and epoxy can interact with water molecules to modulate ice crystal formation and recrystallisation [32,35]. These physical interactions may help reduce mechanical damage to the membrane by limiting ice aggregation around spermatozoa during freezing. The hypothesis we developed regarding how this could occur is presented in Figure 8. The increase in total motility, the preservation of membrane integrity, and the reduction in DNA damage appear to be consistent with reduced physical stress associated with ice formation during the cryopreservation process.

In the literature, the causes of DNA damage during sperm cryopreservation are reported as the alteration of DNA–nuclear protein interactions due to intracellular ice crystal formation [36], the induction of DNA fragmentation through caspase activation and apoptosis-like processes triggered by cryopreservation [37], and oxidative stress [38]. Intracellular ice formation, oxidative stress, and caspase activation can lead to DNA fragmentation; the poly (ADP-ribose) polymerase (PARP) system plays a role in repairing this damage. However, increased stress under cryopreservation conditions can suppress DNA repair mechanisms [39]. The lower DNA damage rate observed across all NGO groups in our study may be related to reduced structural and cellular stress induced by cryopreservation. However, because caspase activation, PARP function, or ROS levels were not directly measured, these mechanisms have not been experimentally verified, and further studies are required.

It is thought that nanoparticle size may be a decisive parameter in this process. Smaller particles have a higher surface area and can form a larger contact surface with water molecules [40]. This situation may enable them to play a more effective role in modulating ice crystal nucleation and aggregation processes. In our study, the observation that 50 nm NGO exerted a protective effect on both motility and plasma membrane integrity, whereas 500 nm NGO exerted a more limited effect, supports the notion that particle size may be an important factor in cryoprotective efficacy.

Despite the study’s important findings, certain limitations should be taken into account. Firstly, the exclusion of markers associated with oxidative stress and cytotoxicity assessments from the study limits in-depth mechanistic interpretation. Additionally, the results of this study are limited to in vitro spermatological parameters and have not been validated by fertility trials or in vivo assessments. Secondly, the sperm samples used in the study were pooled, disregarding individual differences, and therefore, the findings may vary at the individual level. A final limitation is the assessment of DNA integrity. The TB method is widely used and accepted in the literature; however, subjectivity in spermatozoa counting limits its application. Therefore, the use of fluorescent staining methods, such as Annexin V or COMET, in future studies will improve accuracy.

Nevertheless, the present findings demonstrate that NGO can be applied in sperm cryopreservation with measurable benefits, providing a structured experimental basis for future studies designed to integrate mechanistic validation and functional reproductive outcomes.

The use of nanoparticles in the freezing of Angora buck semen may provide significant benefits during freezing and thawing. Studies indicate that various nanoparticles improve motility and preserve acrosome, membrane, and DNA integrity, thereby mitigating cryogenic and oxidative damage [41,42]. However, caution should be exercised when using nanoparticles in sperm thinners because they may have a dual effect [43]. Some nanoparticles (e.g., selenium, cerium dioxide) act as antioxidants [44,45], while others (e.g., silver nanoparticles) may cause excessive ROS accumulation by releasing surface metal ions, activating inflammatory pathways, or disrupting mitochondrial function [46]. Effects may vary depending on the size, shape, surface modification, and dosage of the nanoparticles. In addition, the effects of nanomaterials on spermatozoa also vary depending on the freezing protocol and diluent [47]. In this complex equation, studies revealing the beneficial or toxic properties of carbon-based nanoparticles, such as NGOs, will also contribute to the future development of nano-based sperm diluents and increased success rates in artificial insemination.

## 4. Materials and Methods

### 4.1. Synthesis of NGO Nanoparticles

Unless otherwise stated, the chemicals used in the synthesis were purchased from Sigma-Aldrich (Merck, Darmstadt, Germany).

NGO was synthesized from graphite powders by the Hummers and Offeman method with modifications [48]. First, a specific amount (1 g) of graphite powder was dispersed in 0.5 g of sodium nitrate (NaNO_3_) and sulfuric acid (H_2_SO_4_) (66%, 25 μL) and suspended in an ice bath. Next, potassium permanganate (KMnO_4_, 3 g) was added dropwise to this mixture. The mixture was then sonicated for 20 min in a water bath at approximately 40 °C and subsequently stirred at 500 rpm for approximately 1.5 h until it reached a slurry consistency. Next, 50 mL of water was added to the slurry at room temperature, and the mixture was heated to 95 °C. It was then stirred for 50 min. Subsequently, hydrogen peroxide (H_2_O_2_, 30%, 150 μL) was slowly added to the mixture. This high-temperature mixture was filtered and washed with pure water. The blackish precipitate remaining on the filter was dispersed in water and centrifuged at approximately 1000 rpm for 10 min. This washing process was repeated three times, and the final product was obtained as a powder after drying in an oven.

In general, GO at the micrometer scale was treated with a sonicator, reduced to 500 nm and 50 nm, and produced nano GO (NGO). The core size analysis of the NGO was performed using FE-SEM. The effective diameter was determined by DLS, the surface charge was measured using a zeta potential zetasizer, and the bond vibrations were characterized by FTIR [49].

### 4.2. Animals, Semen Collection, and Sample Preparations

Five healthy 2–3-year-old Angora bucks that had previously sired offspring and had body condition scores of 3–3.5 were used. The animals were housed at the Prof. Dr. Hümeyra Özgen Research and Application Farm at Selçuk University and were fed a balanced ration formulated to meet maintenance and breeding requirements, with free access to water. Semen samples were collected twice a week from late September to mid-October during the breeding season. Over a single 3-week period, semen was collected on 5 separate occasions at 3-day intervals (two collections during the first week, two during the second week, and one during the final week) using an electroejaculator without sedation.

Semen samples were collected from five animals during each semen collection session, and samples with a mass activity score of 3 or higher (1 = lowest, 5 = highest), at least 80% motility, and a concentration of 2.5 × 10^9^ spermatozoa/mL were included in the study. All animals met these criteria, and semen from five animals was pooled in equal volumes at 37 °C to minimize individual variation. As a result, a single semen sample containing sperm from five animals was obtained, and this pooled sample was treated as a single independent biological replicate. This procedure was repeated five times to ensure statistical significance (n = 5). All procedures were carried out in accordance with institutional animal welfare guidelines.

A tris-based egg yolk extender (TEY; 27.1 g/L Trizma, 10.0 g/L fructose, 14.0 g/L citric acid, 20% egg yolk, 5% glycerol, 0.1% antibiotic; pH 6.8, 300 mOsM) was used as the basic extender. Semen was diluted with TEY to a final concentration of 100 × 10^6^ spermatozoa/mL and allocated into experimental groups (Table 1).

The NGO doses used in this study were selected based on previously published studies. At doses ranging from 0.5 to 1 µg/mL, NGO showed a protective effect on pig and mouse sperm, whereas at 10 and 50 µg/mL, it had an adverse effect [50,51]. However, a study conducted on buffalo semen showed that 50 µg/mL NGO provided optimal spermatological parameters [15]. For this reason, in our study, the doses of 10 and 50 μg/mL, which have been reported as negative in some studies and positive in others, were selected to compare the uncertainty in the literature.

### 4.3. Freezing of Sperm

The diluted semen samples were equilibrated at +5 °C for 3 h to allow the interaction of nanoparticles with spermatozoa and to enable membrane phase transitions before freezing. At the end of equilibration, the semen was loaded into 0.25 mL straws in a cooled cabinet at +5 °C, and the ends of the straws were sealed with polyvinyl alcohol powder. The prepared straws were placed horizontally on racks and frozen in liquid nitrogen vapor for 15 min (−20 °C to −60 °C). Frozen semen was then plunged directly into liquid nitrogen (−196 °C) and stored in liquid nitrogen tanks for 3 months [52].

### 4.4. Analysis of Motility and Kinematic Values

Three months after freezing, the straws were removed from liquid nitrogen and thawed in a 37 °C water bath for 30 s for motility analysis. One straw was dissolved for each group in each replication (a total of 25 straws in 5 replications). In cases where technical artifacts or straw-related damages may occur due to the dissolving process, another straw from the same group was dissolved. Sperm samples were transferred to 1.5 mL Eppendorf tubes, and motility parameters were evaluated using a Computer-Assisted Sperm Analysis (CASA) system (AndroVision^®^, Minitube GmbH, Tiefenbach, Germany). Analyses were performed using an Axioscope 5 microscope (Zeiss, Oberkochen, Germany) with a 40× objective, a mobile heating unit set to 37 °C (12057/6000, Minitube GmbH, Tiefenbach, Germany), and a camera specific to AndroVision (12500/4400, Minitube GmbH, Tiefenbach, Germany).

CASA configurations were set as follows: (1) At least 5 different fields (40×) and more than 600 spermatozoa for each sperm sample, (2) 75 frames per second, and (3) Species-specific decision thresholds defined within the AndroVision^®^ system were applied.

For each sample, 7.5 µL semen was dropped on the pre-heated (37 °C) disposable counting chamber with 20 µm depth (12050/0220, Minitube, Tiefenbach, Germany), and the following CASA parameters were measured: (1) total motility (%), (2) progressive motility (%), (3) kinetic parameters [straight-line velocity (VSL, µm/s), curvilinear velocity (VCL, µm/s), average path velocity (VAP, µm/s), distance straight line (DSL, µm), wobble (WOB, VAP/VCL), linearity (LIN, VSL/VCL), straightness (STR, VSL/VAP) [53].

### 4.5. Flow Cytometric Analyses

Flow cytometric analyses included PMAI and MMP assays. FITC-PNA (L7381, Sigma–Aldrich, Burlington, MA, USA) was used for PMAI analyses, JC-1 (T3168, Invitrogen, Carlsbad, CA, USA) for MMP, and Propidium iodide (PI), the primary dye for each analysis, was used.

Frozen sperm was thawed in a 38 °C water bath for 25 s, and a 10 μL sperm sample (5 × 10^6^) was diluted with 490 μL PBS. This step was repeated for each staining method, and then the staining protocol was initiated. For PMAI, 5 μL of FITC-PNA and three μL of PI were added to the prepared sample, and the mixture was incubated in the dark at 37 °C for 30 min. The samples measured by flow cytometry were then counted and divided into three groups: intact membrane–intact acrosome (FITC−PI−, F−P−), intact membrane–damaged acrosome (FITC+PI−, F+P−), and damaged membrane–damaged acrosome (FITC+PI+, F+P+). For MMP, 10 μL JC-1 and 3 μL PI were added to the prepared sample, and the mixture was incubated in the dark at 37 °C in a water bath for 30 min. The samples were then classified and counted by flow cytometry as having high mitochondrial membrane potential (HMMP) and low mitochondrial membrane potential (LMMP) [49].

Analyses were performed using a CytoFlex flow cytometer (Beckman Coulter, Fullerton, CA, USA) with a 488-nm laser and 50 mW of power. Fluorescent emissions were detected using filters at 525 nm, 585 nm, and 610 nm. Cell counting was performed carefully, and an average of 10 × 10^3^ spermatozoa was counted for each sample. The obtained data were evaluated using CytExpert 2.3 software [54].

### 4.6. Analyses of DNA Fragmentation

The toluidine blue (TB) staining method was used to assess DNA fragmentation. Approximately 5 μL of semen/PBS mixture was applied to the slide and dried at room temperature for 30 min. Fixation was performed in acetone at 4 °C for 1 h, and the slides were air-dried. Following a 15 min hydrolysis in 1N HCl at 4 °C, the slides were washed three times for two minutes each in distilled water. After 10 min at room temperature, the slides were stained with 0.05% toluidine blue (pH 4.5), rinsed three times for 2 min each with distilled water, and air-dried. Under a light microscope (DM2000, Leica, Wetzlar, Germany), at least 200 spermatozoa were randomly counted in ten distinct fields for each plate. These spermatozoa were then classified as either aberrant TB (−) or regular TB (+). The heads of sperm cells with low chromatin integrity were dark violet (purple), while those with high integrity were pale blue [55].

### 4.7. Statistical Analysis

For statistical analysis of the study data, IBM SPSS Statistics 26 was used. The data’s conformity to a normal distribution was assessed using the Shapiro–Wilk test and histogram plots. The homogeneity of variances was evaluated using Levene’s test. For heterogeneous comparisons, the Welch statistic was considered. The means of post-thaw spermatological parameters and imaging results for each experimental group were determined using one-way analysis of variance (ANOVA). For the same parameters, comparisons between groups with significant differences were performed using Duncan’s multiple-comparison test. A significance level of *p* < 0.05 was considered statistically significant.

## 5. Conclusions

In conclusion, the NGO shows promise as a potential cryoprotectant agent to improve the cryopreservability of Angora buck sperm under in vitro conditions. In particular, the NGO at 50 nm in size and 50 µg/mL in concentration yielded the most favorable results for motility, membrane and acrosome integrity, and DNA integrity parameters. However, as the present findings are restricted to post-thaw in vitro sperm quality parameters, and the impact of NGO supplementation on fertilization success and reproductive outcomes remains to be determined through well-designed in vivo studies. Therefore, these findings should be considered preliminary and require further validation before definitive conclusions can be drawn.

## Figures and Tables

**Table 1:** Experimental groups and extender contents.

Groups	Extender Content
Control	TEY extender
NGO 50-10	TEY extender + 50 nm and 10 μg/mL NGO
NGO 50-50	TEY extender + 50 nm and 50 μg/mL NGO
NGO 500-10	TEY extender + 500 nm and 10 μg/mL NGO
NGO 500-50	TEY extender + 500 nm and 50 μg/mL NGO

## Data Availability

Research data from the study are available from the corresponding authors upon request.

## Abbreviations

The following abbreviations are used in this manuscript:

ART	Assisted Reproductive Technologies
CASA	Computer-Assisted Sperm Analysis
DLS	Dynamic Light Scattering
DMSO	Dimethyl Sulfoxide
DNA	Deoxyribonucleic Acid
FE-SEM	Field Emission Scanning Electron Microscopy
FITC-PNA	Fluorescein Isothiocyanate–Peanut Agglutinin
FTIR	Fourier-Transform Infrared Spectroscopy
GO	Graphene Oxide
HAC	Head Activity
HMMP	High Mitochondrial Membrane Potential
JC-1	5,5′,6,6′-Tetrachloro-1,1′,3,3′-Tetraethylbenzimidazolylcarbocyanine Iodide
LMMP	Low Mitochondrial Membrane Potential
MMP	Mitochondrial Membrane Potential
NGO	Nano-Graphene Oxide
PBS	Phosphate-Buffered Saline
PI	Propidium Iodide
PMAI	Plasma Membrane and Acrosome Integrity
PUFA	Polyunsaturated Fatty Acid
ROS	Reactive Oxygen Species
SEM	Standard Error of the Mean
SSC-A	Side Scatter Area
FSC-A	Forward Scatter Area
FSC-H	Forward Scatter Height
TB	Toluidine Blue
TEY	Tris-Based Egg Yolk Extender
VAP	Average Path Velocity
VCL	Curvilinear Velocity
VSL	Straight-Line Velocity
WOB	Wobble
LIN	Linearity
STR	Straightness
